# A complex ePrescribing antimicrobial stewardship-based (ePAMS+) intervention for hospitals: mixed-methods feasibility trial results

**DOI:** 10.1186/s12911-024-02707-9

**Published:** 2024-10-11

**Authors:** Christopher J. Weir, Susan Hinder, Imad Adamestam, Rona Sharp, Holly Ennis, Andrew Heed, Robin Williams, Kathrin Cresswell, Omara Dogar, Sarah Pontefract, Jamie Coleman, Richard Lilford, Neil Watson, Ann Slee, Antony Chuter, Jillian Beggs, Sarah Slight, James Mason, David W. Bates, Aziz Sheikh

**Affiliations:** 1 Edinburgh Clinical Trials Unit, Usher Institute, University of Edinburgh, Edinburgh, UK; 2 Usher Institute, University of Edinburgh, Edinburgh, UK; 3 Newcastle Upon Tyne Hospitals NHS Foundation Trust, Newcastle, UK; 4 Institute for the Study of Science, Technology and Innovation, University of Edinburgh, Edinburgh, UK; 5 Department of Health Sciences, University of York, York, UK; 6 Institute of Clinical Sciences, University of Birmingham, Birmingham, UK; 7 University of Birmingham, Birmingham, UK; 8 School of Pharmacy, Newcastle University, Newcastle, UK; 9 Warwick Medical School, University of Warwick, Warwick, UK; 10 Harvard Medical School, Boston, USA

**Keywords:** Health informatics, Bacteriology, Infectious diseases, Microbiology, Decision support

## Abstract

**Background:**

Antibiotic resistant infections cause over 700,000 deaths worldwide annually. As antimicrobial stewardship (AMS) helps minimise the emergence of antibiotic resistance resulting from inappropriate use of antibiotics in healthcare, we developed ePAMS+ (ePrescribing-based Anti-Microbial Stewardship), an ePrescribing and Medicines Administration (EPMA) system decision-support tool complemented by educational, behavioural and organisational elements.

**Methods:**

We conducted a non-randomised before-and-after feasibility trial, implementing ePAMS+ in two English hospitals using the Cerner Millennium EPMA system. Wards of several specialties were included. Patient participants were blinded to whether ePAMS+ was in use; prescribers were not. A mixed-methods evaluation aimed to establish: acceptability and usability of ePAMS+ and trial processes; feasibility of ePAMS+ implementation and quantitative outcome recording; and a Fidelity Index measuring the extent to which ePAMS+ was delivered as intended. Longitudinal semi-structured interviews of doctors, nurses and pharmacists, alongside non-participant observations, gathered qualitative data; we extracted quantitative prescribing data from the EPMA system. Normal linear modelling of the defined daily dose (DDD) of antibiotic per admission quantified its variability, to inform sample size calculations for a future trial of ePAMS+ effectiveness.

**Results:**

The research took place during the SARS-CoV-2 pandemic, from April 2021 to November 2022. 60 qualitative interviews were conducted (33 before ePAMS+ implementation, 27 after). 1,958 admissions (1,358 before ePAMS+ implementation; 600 after) included 24,884 antibiotic orders.

Qualitative interviews confirmed that some aspects of ePAMS+ , its implementation and training were acceptable, while other features (e.g. enabling combinations of antibiotics to be prescribed) required further development. ePAMS+ uptake was low (28 antibiotic review records from 600 admissions; 0.047 records per admission), preventing full development of a Fidelity Index. Normal linear modelling of antibiotic DDD per admission showed a residual variance of 1.086 (log-transformed scale). Unavailability of indication data prevented measurement of some outcomes (e.g. number of antibiotic courses per indication).

**Conclusions:**

This feasibility trial encountered unforeseen circumstances due to contextual factors and a global pandemic, highlighting the need for careful adaptation of complex intervention implementations to the local setting. We identified key refinements to ePAMS+ to support its wider adoption in clinical practice, requiring further piloting before a confirmatory effectiveness trial.

**Trial registration:**

ISRCTN Registry ISRCTN13429325, 24 March 2022.

**Supplementary Information:**

The online version contains supplementary material available at 10.1186/s12911-024-02707-9.

## Background

Worldwide, antibiotic resistant infections are responsible for over 700,000 deaths per annum [1]. In healthcare, contributing factors to the development of antimicrobial resistance (AMR) include the continuing inappropriate use of antibiotics through high levels of prescribing and administration [2]. It is therefore essential to optimise antimicrobial stewardship (AMS) with regards to the selection, optimal dose and duration of antibiotic prescriptions to minimise the development of AMR [3].

European surveillance data on antibiotic consumption identified that high in-hospital use of antibiotics is prevalent in the United Kingdom (UK) and that hospital prescribing increased by 6.3% in the period 2016 − 19 [4]. The substantial impact of the first waves of the severe acute respiratory syndrome coronavirus 2 (SARS-CoV-2) pandemic [5] exacerbated this trend: inpatient antibiotic usage per hospital admission in England rose by 10.6% in 2019–20, with systematic review evidence suggesting an increase in inappropriate use [6].

In recognition of the risks arising from AMR, Public Health England (now the UK Health Security Agency) released guidance promoting a "Start Smart—Then Focus" strategy among practitioners for antibiotic initiation and maintenance [7, 8]. Furthermore, a "reduction in antibiotic use per 1,000 admissions" is expressly targeted by National Health Service (NHS) England Antimicrobial Resistance and Antimicrobial Stewardship Commissioning for Quality and Innovation (CQUIN), a framework intended to support quality improvement in healthcare [9].

Electronic prescribing (ePrescribing) systems, often referred to as computerised provider order entry (CPOE) with clinical decision support (CDS) systems, now represent a key tool in managing AMS in line with the above policies [10–12]. The impact of CDS guidance can be extended using techniques that support clinicians and hospitals in prioritising AMS by, for example, facilitating timely antibiotic prescription review [11].

In response to the urgent clinical need and the potential of ePrescribing systems to contribute to a solution to AMR, we initiated a research programme to plan and develop an ePrescribing-based Anti-Microbial Stewardship complex intervention (ePAMS+). In the hospital in-patient setting, ePAMS+ combines a CDS tool embedded within an ePrescribing and Medicines Administration (EPMA) system with educational, behavioural, and organisational elements to support effective implementation of antibiotic prescribing that is consistent with local guidelines informed by AMR rates.

Following the development of ePAMS+ , we intended to confirm its effectiveness and cost-effectiveness in a full-scale clinical trial. In order to inform the design of that confirmatory study, we undertook a feasibility trial [13] to investigate the implementation and acceptability of the ePAMS+ intervention and to test data extraction processes. Here, we report the feasibility trial findings.

## Methods

The protocol of the feasibility trial, including full details of the ePAMS+ intervention, has been published [13]. Here, we summarise the key features of the study protocol, namely the trial design, intervention, and the qualitative and quantitative components of this mixed-methods research. Table 1 outlines the 10 feasibility trial objectives and the method used to address each one.

**Table 1:** Feasibility trial objectives and summary findings

Objective	Methodology	Completed	Key findings
1. Explore acceptability to users of the content and any barriers to uptake of the ePAMS+ technical component	Qualitative	Yes	Users reported difficulty in finding review documentation
2. Investigate whether ePAMS+ Antibiotic Order Plans can be used as intended in clinical practice and the reasons why this may not happen	Qualitative	Yes	Used correctly, but infrequently due to it being non-compulsory. ePAMS+ did not cover non-standard cases such as combinations of antibiotics
3. Assess acceptability to healthcare professionals of the ePAMS+ intervention organisational and educational components	Qualitative	Yes	Champion appointed and led training. No regular, formalised meetings undertaken; generally low awareness among staff Online training – reduce duration by removing video content; staff welcomed “at elbow” on the job support and training
4. Establish how ePAMS+ may best be implemented in various care contexts and health information systems	Qualitative	Yes	Assessment Suite ward at start of many patients’ admission journey identified as a key point for implementing ePAMS+ . Recognised importance of use in general medical wards, not just those in which specialist microbiology input is available
5. Confirm whether the processes used to implement ePAMS+ are acceptable and feasible	Qualitative	Yes	Found not to be acceptable or feasible; points towards opportunities for greater communication and improvements to the implementation plan
6. Create a Fidelity Index to measure the extent to which the core ePAMS+ intervention themes are delivered in antibiotic prescribing and test usability of the Fidelity Index	Quantitative	Partially	The routine data extract can inform whether critical decision-making points (or core ePAMS+ intervention themes) were adhered to when delivering ePAMS+ intervention. Insufficient antibiotic review data to progress further with Fidelity Index development
7. Explore hypothesised mechanisms of action, refine programme theory and select appropriate process analysis measures to be used a future trial evaluating ePAMS+	Qualitative	Yes	The trial generated hypotheses which will require further testing, for example regarding alternative delivery of education component
8. Confirm whether ePAMS+ can be integrated successfully into hospitals to facilitate prescribing behaviour changes	Qualitative / Quantitative	Yes	Full integration not achieved, evidenced by low uptake of online training and small number of reviews undertaken. Importance of intervention being optional rather than mandatory
9. Build processes of collecting outcome data from EPMA systems before and after introduction of the ePAMS+ ePrescribing tools	Quantitative	Partially	Some outcomes could be successfully extracted and derived from EPMA system. Some process measures could only be recorded after ePAMS+ had been implemented. Lack of availability of indication data meant that a number of outcomes could not be derived
10. Quantify between-patient variability in total antibiotic consumption, confirming the sample size required for a full-scale trial evaluating the ePAMS+ intervention	Quantitative	Yes	Antibiotic consumption data modelled and residual variance used to inform sample size calculations for future ePAMS+ evaluation studies

### Trial design

This trial was a mixed-methods evaluation of the implementation of the ePAMS+ service-level complex intervention, involving the collection of qualitative and quantitative data before and after ePAMS+ was introduced. We planned to include two hospital Trusts (a Trust being a hospital or group of hospitals in England’s NHS) which had adopted the widely-deployed Cerner Millennium EPMA system and to sample wards purposively within each to ensure that intervention feasibility would be investigated across a range of clinical scenarios.

During trial planning it emerged that Cerner EPMA system within one of the recruited NHS Trusts included mature local adaptations, developed during the SARS-CoV-2 pandemic, which overlapped with some aspects of the ePAMS+ intervention technical tool. We therefore proceeded in one NHS Trust encompassing two hospital sites, and increased the number of wards included in each to maximise the variety of clinical contexts covered by the trial. We studied the acute assessment suite and wards providing care for a wide range of conditions and patient groups i.e., general medical, respiratory, gastroenterology, hepatology, stroke, care of the elderly, gynaecology, infectious diseases, haematological cancers and solid tumours.

The participating NHS Trust provided acute, specialist and community services. It served a population of over three million and had 1,800 beds, 16,000 staff and 1.84 million patient contacts per year. It had a Care Quality Commission rating of Outstanding at the time of the study. It was a Global Digital Exemplar (rated by the Healthcare Information and Management Systems Society (HIMSS) as stage 6 (of 7), indicating a high level of digital maturity.

### Intervention

The ePAMS+ intervention consisted of three interrelated components: a technical tool embedded within the EPMA system; an organisational and behavioural change model; and an AMS online training module.

The technical tool incorporated the following components in the Cerner EPMA system:

1. *antibiotic order plans* for antibiotic prescribing and scheduling of a series of review points at which prescription changes may be necessary (for example, an order plan for amoxicillin may specify Dose “1 g”, Drug form “Capsule”, Route of administration “Oral”, Frequency “Three times a day”; and schedule a review point at which cultures would be reviewed and a decision made on stopping, continuation or switching);
2. *decision aid* to record the certainty of the original prescriber’s decision regarding the requirement for antibiotics, to help inform any subsequent decision to end a prescription (based on the ARK classification [14] “possible infection risk”, “probable infection” or “finalised diagnosis of infection” and including an additional category, “prophylaxis”);
3. *decision aid* recording expected site of infection (body system, selected from a drop-down list) and working diagnosis (indication, using a free text description);
4. *information pages* to help users make the most of ePrescribing tools, including, for example, user guides to *antibiotic order plans*, diagnostic confidence *decision aid* and *ward lists* of patients receiving antibiotics;
5. *prompts* for antibiotic review by prescribers; and
6. *links* from the review screen to microbiology and/or pathology data.

Use of the ePAMS+ technical tool for antibiotic prescribing was, by necessity, optional rather than mandatory, since a mandatory roll-out would have impacted on prescribing in paediatrics and outpatients, both of which were outside the scope of this feasibility trial.

The behavioural and organisational support component of ePAMS+ was arranged as follows: in each hospital enrolled in the feasibility trial, an “ePAMS+ Champion” planned to assemble a local implementation team. This team promoted ePAMS+ usage, for example, during grand rounds, departmental or specialty team meetings, clinical governance sessions and training courses for junior doctors, nurses and pharmacists.

In the final component of the ePAMS+ intervention, prescribers, pharmacists and nurses working on participating hospital wards were encouraged to complete the ePAMS+ online eLearning training module within the Health Education Learning Management (HELM) system, a training platform widely used in the NHS. The module content covered the principles of AMS and the tools provided by the ePAMS+ intervention. Progress of participants was recorded through pre- and post-module tests comprising of multiple choice single best answer questions based on the learning outcomes.

Further in-depth information on the details of the ePAMS+ intervention has been provided, using the Template for Intervention Description and Replication (TIDieR) checklist [15], in the feasibility trial protocol [13] and a more detailed intervention development publication [16].

### Qualitative methods

We conducted a combination of longitudinal semi-structured qualitative interviews and non-participant observations before and after the introduction of ePAMS+ in two hospitals of the same Trust where the intervention was implemented.

### Sampling and recruitment

Our sampling strategy was purposeful, seeking to include prospective and current users of the ePAMS+ interventions. We recruited pharmacists and doctors involved in prescribing and reviewing of antibiotics in participating wards from a range of demographics, levels of seniority and specialties, including general medicine, microbiology, and infectious diseases. Nurses were also interviewed. Staff were recruited directly via recommendation of a senior ward clinician or through recruitment leaflets distributed to wards. The research team gave potential staff participants written project information summarising what participation would involve. They were given at least 24 h to consider their decision and were free to withdraw at any time. On receiving a completed consent form, a researcher arranged a suitable time with the participant for the interview. Recruitment during the site visits of the two hospitals stopped when we reached data saturation (the point when no new themes emerged in concurrent analysis) [17].

### Data collection

We explored existing work practices before the introduction of the intervention and investigated changes to these after the introduction of the new system. The interview content was developed specifically for this study [see Supplementary Material 1 and [13] for details of the topic guide]. Interviews were audio-recorded and transcribed verbatim by a professional transcription service.

Observations included: two grand rounds with consultants and junior doctors (each lasting 60–90 min); clinician discussions (lasting 30–45 min) before and after each grand round; two handover meetings at change of shift in the assessment suite ward doctors’ office (lasting 30 min each); and usage of the ePAMS+ technical tool by five clinicians (lasting 5–30 min). The researcher took notes during the observations, which were later transcribed together with the researcher’s reflections. Observations were undertaken opportunistically as and when potential participants were available and willing to be observed.

### Qualitative analysis

Data collection and analysis were iterative, allowing developing themes to be investigated further and contrasting evidence to be sought. Transcripts were coded by one researcher (SH) using NVivo (QSR International, V12) and discussed with two senior researchers (RW, KC) to confirm emerging themes. Notes from observations were analysed using the coding framework developed during the analysis of the interview transcripts and used as contextual information to understand the setting of the implementation and use of ePAMS+ .

Thematic data analysis investigated perceptions of the intervention and any modifications required to improve ePAMS+ and its implementation (Objectives 1–5). We also identified probable mechanisms of action to be explored in a process evaluation (Objective 7) embedded in a potential future trial of ePAMS+ .

The thematic analysis applied deductive and inductive approaches [18, 19]. The deductive aspect involved preparing a coding structure, based on our previously-developed Technology, People, Organisations and Macroenvironmental factors (TPOM) evaluation framework [20] covering technology, work practices, organisational factors and wider macro-environmental considerations. Tensions, challenges and variations in participant views and experiences over time were focal points of analysis.

### Quantitative methods

Patients eligible for inclusion were those aged ≥ 16 years admitted to hospital as a medical inpatient who had an antibiotic prescription flagged or an antibiotic order plan started within the EPMA system. As ePAMS+ was a service-level intervention, all eligible admissions to participating wards were included.

As a feasibility study, no formal sample size justification was required; we aimed to study at least 100 admissions per ward to enable precise estimation of between-patient variability, by ward and overall, in antibiotic use (Objective 10). This number of admissions also allowed exploration of the feasibility of data extraction across a wide range of clinical presentations.

The local NHS Trust Pharmacy Informatics Team obtained data extracts by running a standardised data query on the EPMA system. These queries were run prior to activation of the ePAMS+ intervention and after implementation. We extracted data (Table 2) to derive outcomes for quantitative analysis purposes (Objectives 9,10), such as total antibiotic use and its variability between patients; and as process measures to help understand how the ePAMS+ system was being used (Objective 8). Data extracted from participating NHS Trusts were transferred via secure file transfer protocol (Serv-U FTP) to the National Safe Haven maintained by Public Health Scotland.

**Table 2:** Co-primary outcomes, secondary outcomes and process measures

Outcome	Data extracted successfully
**Co-primary outcomes**	
Antibiotic defined daily dose per admission	✓
30-day mortality	✓
**Secondary outcomes**	
Length of hospital stay	✓
Days of therapy	✓
Days of IV therapy	✓
Diagnostics	✓
Number antibiotics prescribed	✓
Number antibiotic courses	✓
Repeat courses for same indication	✘
Number courses for same indication	✘
Switch of frequency	✘
Switch of dose	✘
IV to oral switch	✓
Oral to IV switch	✓
Switch to alternative antibiotic	✓
Switch to broad spectrum	✓
Discontinuation of therapy	✓
Courses concordant with local guidelines for antibiotic choice	✘
Courses concordant with local guidelines for antibiotic duration	✘
Resistance rates	✘
Susceptibility	✘
Acquisition of multi-drug resistant organism	✘
Healthcare-associated infection	✘
Episodes of *Clostriodes difficile*	✘
Episodes of methicillin-resistant *Staphylococcus aureus*	✘
Episodes of gram-negative *Bacilli*	✘
**Process measures**	
Clinical decision support – workaround	✘
Clinical decision support – alert frequency	✘
Clinical decision support – alert override	✘
Clinical decision support – use of CDS order set	✓
Time to administration	✓
Time to active therapy (first dose)	✓
Time spent prescribing	✘
Documentation of indication	✘
Documentation of duration	✓
Documentation of stop	✓
Documentation of review	✓
Documentation of decision-making	✓
Switch from Reserve to Watch group antibiotic	✘
Switch from Watch to Reserve group antibiotic	✘
Adherence to clinical guidelines	✘
Adherence to documented sensitivity	✘
Appropriate dose for indication	✘

*IV* Intravenous

All data were held in a project-specific area in the National Safe Haven with access limited to named project researchers via a unique username and multi-factor authentication. The National Safe Haven reviewed all outputs to ensure these would not disclose the identity of any participant.

Site staff ePAMS+ training information was captured on the HELM system to assess uptake of training (recording professional discipline, date/time of module completion, time spent on learning and pre- and post-test scores). Data were anonymised at site prior to transfer to the research team.

The quantitative component of the feasibility trial primarily aimed to 1) determine the ability to derive total antibiotic consumption, measured as the number of defined daily doses (DDD) per admission, and to estimate the variability in this outcome; 2) confirm the ability to capture complete data from local Cerner EPMA system configurations; and 3) verify the ability to measure defined outcomes such as mortality at 30 days post-admission. DDD per admission and mortality at 30 days post-admission will be co-primary outcomes in any future full-scale evaluation of the ePAMS+ intervention. Table 2 lists the secondary outcomes and process measures for which feasibility of data extraction was assessed. It was not an objective to examine intervention effects in this feasibility trial and the frequency and nature of data extracts gave no scope to monitor adverse event occurrence in real time.

### Quantitative analysis

Although there was no randomisation or concealment of the intervention in this service-level feasibility study, the statistical analysis plan was pre-specified before any study data were recorded.

Following descriptive summaries of total antibiotic consumption, overall and by hospital site, the between-patient variability in total antibiotic consumption, measured as the number of DDD per admission, was planned to be quantified (Objective 10) using a normal linear model (following log-transformation if necessary). Ward type was included in the model so that its component of variance could be estimated. Season (according to UK Met Office classification [21]) and an indicator variable for ePAMS+ intervention implementation were also considered as model factors. Separate summaries were also reported for antibiotic subtypes: intravenous, oral, broad spectrum and narrow spectrum.

Other quantitative outcomes were assessed using two criteria. First, we determined whether it was possible to derive each outcome listed in Table 2 from the EPMA system data extract (Objective 9). Secondly, we summarised outcomes descriptively, including the rate of missing data, overall and by site and by ward. Objective 8 (successful ePAMS+ integration into care) was addressed by the level of data gathered on relevant outcomes, such as the number of antibiotic reviews which took place.

### Fidelity index

Assessing fidelity helps increase confidence that changes in outcomes are attributable to the intervention and that behavioural interventions were implemented as described in the protocol [22, 23]. For Objective 6 we aimed first to develop a Fidelity Index to measure the degree to which the key ePAMS+ elements were delivered in practice, and secondly to determine the usability of the index.

As outlined more fully in the protocol [13], the Fidelity Index development addressed three aspects, supported by data extracts from the Cerner EPMA system: (1) identification of critical decision-making points for prescribers; (2) building understanding of the data structure to enable fidelity to be coded automatically; and (3) devising a cumulative score to quantify fidelity across all of the critical decision points.

Within the feasibility trial, we then aimed to pre-test the automation and derivation of the Fidelity Index.

### Patient and public involvement

Throughout the development and delivery of the feasibility trial, our two patient and public representatives (AC, JB) offered guidance and feedback on decisions in the monthly programme management group meetings.

### Ethical considerations and reporting guidelines

Patient informed consent was neither required nor sought in this feasibility trial. The implementation pack did however contain a patient information leaflet to help healthcare staff explain the process of antibiotic use and review to patients. Patients admitted to participating wards did not have the opportunity to opt out of the use of their routine de-identified administrative data covering the measures outlined in Table 2.

This trial report follows the checklist items from the CONSORT reporting guidance extension for pilot and feasibility studies [24] which are relevant to a non-randomised feasibility trial.

## Results

This research took place from April 2021 to November 2022. The SARS-CoV-2 pandemic therefore impacted substantially on the trial, not only due to the resulting backlog of NHS Research and Development approvals which delayed commencement, but also on the context of the intervention implementation, resulting from changes in healthcare provision and developments in AMS which were accelerated during the pandemic response.

### Qualitative data collected

We carried out a total of 60 interviews, 33 prior to intervention implementation (18 video or telephone, 15 face-to-face) and 27 over two site visits around four weeks and seven weeks after implementation. Each interview lasted between three and 60 min. 18 participants were interviewed during the first post-implementation visit, nine during the second visit, and three were interviewed at both visits.

Twenty-two females and 35 males were interviewed across a range of grades: four pharmacists, one nurse, 19 early (foundation years 1&2) trainees, 10 later (years 3–7) internal medicine or specialty clinical trainees, 10 registrars and 13 consultants.

### Implementation context

Five wards prescribing large volumes of antibiotics were selected first for implementation and qualitative fieldwork (infectious diseases, oncology, haemato-oncology, care of the elderly and respiratory diseases). These wards had rigorous oversight of antibiotic prescribing and reviewing, and close communication with microbiologists.

During the fieldwork, the study lead consultant and ePAMS+ champion advised including the assessment suite (a ward holding patients after the emergency department, where decisions are made whether to admit patients to the hospital or discharge them home) in the qualitative evaluation, due to the large volume of antibiotics prescribed there. The use of ePAMS+ for prescribing antibiotics was not compulsory and clinicians could also use the usual ‘Medications’ list in the Cerner EPMA system to prescribe antibiotics.

At the time of this evaluation, the NHS was experiencing what were widely regarded as the worst pressures it had experienced in its 70-year history, with a severe shortage of nursing and medical staff. Consequently, shortly after the ePAMS+ intervention was launched, the Trust declared an Operations Pressures Escalation Level (OPEL) [25] level 4 emergency on 20 October 2022, this highest rating indicating that it was unable to provide comprehensive care and patient safety was considered to be at very high risk. In the week before and the week after this date the hospital had declared OPEL 3 emergencies, indicating high clinical risk. This context was reflected in the number of clinicians who reported prescribing when they were tired and under pressure. It also affected the introduction and use of ePAMS+ : training and engagement with staff did not take place as planned. None of the clinicians interviewed had undertaken the online training, which is likely to have impacted knowledge and use of the system.

Further detailed quotations on the following themes may be found in Supplementary Table S1.

### Promoting antibiotic review

The focus of ePAMS+ was the antibiotic review function to promote AMS. Participants perceived ePAMS+ to promote good AMS practice and prescribers considered the steps involved in entering information gave them an opportunity to consider their prescribing decisions.

*“… they [prescribers] are busy in different ways and I think in those instances it might be useful just to have that reminder and that can be a moment where you can actually just think, is this the right antibiotic…there's definitely a good case for it”. (Participant 3, junior doctor, foundation year 2)*

The ePAMS+ antibiotic review function was intended to create a new workflow, with the aim of improving antibiotic prescribing practice through implementation of the logic model summarised in the intervention development [16] and addressing identified issues relating to the selection, optimal dose and duration of antibiotic prescriptions [3, 16]. However, at this early stage of implementation, few people had used ePAMS+ and it was unclear to what extent the antibiotic review function had actually interrupted existing workflows. When used on selected wards, such as infectious diseases and haematology/oncology, the review function was considered useful in structuring and formalising the review process. The review documentation on ePAMS+ made formal and explicit what was previously informal and implicit.

*“The ePAMS system lets you document things as you’re going along, so you might have a senior review but you might not have the blood culture results back yet and various things like that. So, it does provide a framework for ticking those things off, as it were…” (Participant 1, consultant)*

Clinicians were open about the fact that they sometimes forgot antibiotic review and appreciated the prospect of the visual reminders and prompts associated with ePAMS+ functionality.

The antibiotic review function was also perceived to help mitigate the effect of different clinicians being involved in an individual patient pathway. It was a reminder to a clinician who had not originally prescribed the antibiotic and ePAMS+ was viewed to effectively bring the patient’s antibiotic journey together in one place.

*“I think it’s definitely great in that it prompts you to do a medication review … with our rota, where there’s not always that continuity of juniors covering the same service each week or even day to day...” (Participant 8, junior doctor, foundation year 2)*

### Training and launch of ePAMS+ 

Due to time pressures in the participating NHS Trust, the four lead clinicians in the study were not able to prepare the launch for ePAMS+ as originally planned. Subsequently, the launch was communicated verbally and training was delivered ad hoc by the lead consultants. There were no notices on the wards to announce either the ePAMS+ ‘go-live’ to prescribers, or the availability of the online training programme.

A clinical informatics trainee clinician commented on the significant effort that would be required in making all clinicians aware of any changes in the prescribing system. He later became involved in the ad hoc training of clinicians on ePAMS+ .

*“…it's a lot of work…to raise awareness and encourage clinicians to use it. And then once you do that, you still need to redo the awareness project every few weeks, really, on the assessment suite, as trainees change every few months… (Participant 5, specialty trainee, year 5)*

None of the staff interviewed said they were aware of the 30-min online training module developed by the ePAMS+ research team. Discussion of this surfaced doubts over whether a video would be the most effective way of training clinicians to use ePAMS+ . Practical ‘at elbow’ training was considered more valuable, as it would be more closely linked to practice. Some suggested it could be incorporated into existing teaching activities.

Some staff had been taught how to use ePAMS+ by colleagues on the same ward. However, in such a busy environment the ‘training’ was incomplete and there was little time available for the clinician to understand the full ePAMS+ functionality.

It became apparent during this early implementation period that the best place to focus training and encourage the use of ePAMS+ was the 50-bed assessment suite as many patients were prescribed antibiotics there, or in Accident & Emergency, before being admitted to a hospital ward. If admitted, the antibiotic review alert would then appear in the patient’s electronic health record 48 h later when a different clinician would be responsible for carrying out the review.

*“I think it’s more important that it happens here, as in acute medicine, because you want the review to happen after a couple of days of admission when you’ve got results available.” (Participant 20, specialty trainee, year 1)*

### ePAMS+ user experience

Changing to a new system takes time and effort, and clinicians who were tired and busy were more likely to forget to use ePAMS+ , especially as it was not compulsory at this stage.

The design of ePAMS+ was not perceived as being intuitive. For example, ePAMS+ was not found on the usual Medications list of the Electronic Patient Record (EPR); it was located in a separate place under the Request and Care Plans list. However, most clinicians felt ePAMS+ was something they would get used to and that it did not negatively interrupt the workflow of prescribing antibiotics.

*“It’s no more bother for me to prescribe it via ePAMS than to prescribe it normally…it’s just as easy, and then if it helps further down the line to stop inappropriate use of antibiotics…” (Participant 15, speciality trainee, Year 2)*

An important goal of the feasibility trial was to identify aspects of the design of ePAMS+ that required further development. Feedback from user experience identified several challenges and opportunities for system enhancement that had not been recognized in earlier co-development workshops with stakeholders.

In the case of an immediate single dose of an antibiotic, the 48-h prompt for review was not perceived to be relevant and the lack of order sets on ePAMS+ was considered an inconvenience and a risk to patient safety.

*“… order sets…I’ve just found them quite helpful in that they prompt you…I think ePAMS would be really good if ePAMS had order sets in the same way.” (Participant 10, junior doctor, foundation year 2)*

One of the consultants acknowledged that the default doses of antibiotics on ePAMS+ needed to be adjusted because they were not commonly used. Also, ePAMS+ would not allow the prescribing of two antibiotics without exiting and re-entering the system, which was considered a risk to patient safety because interruptions in the clinician’s workflow may result in the second antibiotic not being prescribed.

*“The only difficulty is if you need to prescribe, say, two antibiotics at once, like amoxycillin and clarithromycin, you’ve got to do one, sign it off, and then do the other. For some reason, it won’t let you select two at once.” (Participant 19, specialty trainee, year 3)*

Altering an antibiotic prescription was also not viewed to be straightforward and created more work for the clinician.

Finally, ePAMS+ included commonly prescribed antibiotics but did not list all antibiotics, which made it difficult to prescribe combinations.

### Integration of ePAMS+ with multidisciplinary ways of working

AMS includes many different healthcare professions within the organisation. For example, we found that the nursing role is critical to AMS. Although nurses did not prescribe in this area (on the whole) and would not be able to action the Antibiotic Review prompt, they were often more aware of the patient’s antibiotic status than doctors due to their day being structured around drug rounds. We found they were also highly motivated to move a patient from intravenous to oral routes. Intravenous antibiotics involved two nurses preparing and administering the antibiotic and there was considerable work involved in preventing infection of the tubing and dealing with cannulas coming out of place. In contrast, administration of an oral antibiotic took only one nurse and was less time-consuming.

*“It’s really time-consuming doing IV, intravenous. It can be detrimental to the patients because they’ve got…you’re going into a vein, there’s risks of getting infections from cannulas or midlines so there is a risk to that and it’s more intervention than what you would do taking an oral medication.” (Participant 5, senior nurse)*

Microbiologists were also an important part of AMS in providing support to both junior doctors and consultants on the choice of antibiotics when patient conditions were complex and the guidelines lacked sufficient information. The wards selected for the early implementation of ePAMS+ prescribed antibiotics in large volumes and already had a close relationship with microbiologists. The infectious diseases ward had a particularly close relationship as junior doctors were training in both infectious diseases and microbiology.

Another area where cross-disciplinary relationships were important was with pharmacists. At the time of the study, there were no pharmacists specialising in AMS employed by the Trust. Two interviewees acknowledged the potential for greater involvement of ward pharmacists in AMS. Although the pharmacists checked patient medications against general practitioner records, screened for allergies and reviewed compatibility of prescribed medications, they were not directly involved in implementing ePAMS+ . Since the feasibility trial completed, the Trust has appointed two new specialist AMS pharmacists.

### Quantitative evaluation

Cerner EPMA system data extraction commenced on 1 September 2022 and continued until admissions on 7 November 2022. Data collection ended at this point because a sufficiently large number of admissions had been recorded to address the quantitative objectives. For logistical reasons the ePAMS+ intervention was activated across participating wards in a staggered manner between 5 October 2022 and 7 November 2022.

Table 3 illustrates the numbers of admissions and patients by study period. In total, 24,884 antibiotic orders were recorded across 14 wards. Approximately equal numbers of women and men were included: 706 admissions of females and 652 of males (52%/48%) were included in the period before the ePAMS+ intervention and 321 (54%) and 279 (46%) after the ePAMS+ intervention. Median age was similar in the periods pre- and post-intervention (71 and 72 years, respectively).

**Table 3:** Number of admissions and patients by study period

	**Before ePAMS+ **	**After ePAMS+ **
Number of admissions	1358	600
Number of completed admissions	1256	587
Number of patients	1267	501
Number of admissions with 30 days of follow-up at data cut-off^a^	1323	336

^a^*N* = 299 admissions had not completed follow-up for the 30-day mortality outcome at the point of data lock and final reporting

The findings on developing processes for extracting outcome data from EPMA systems (objective 9), are summarised in Table 2. This lists the co-primary and secondary outcomes and process measures from the study protocol [13], annotated according whether or not it was possible to derive data on each. Approximately half of the outcomes considered could be extracted automatically (20 of 43).

Notable omissions included documentation of indication. Indication was embedded within a free text data item in the EPMA system, and this free text was considered by information governance colleagues to be inappropriate for transfer to the safe haven for analysis due to the risk it contained disclosive information. It was not therefore possible to report on the number of courses by indication, whether any repeat courses were administered for an indication and whether the dose was appropriate for the indication. Data on key infections such as episodes of *Clostridiodes difficile*, methicillin-resistant *Staphylococcus aureus* (MRSA) and gram-negative *Bacilli* were also unavailable for the same reason. A lack of clinical guidelines or reserve/watch lists in the data extract meant that outcomes relying on those could not be derived.

Some process measures (use of clinical decision support order set; documentation of duration) could be recorded only during the period when the ePAMS+ intervention was switched on. The review process was rarely documented (objective 8): only 16 of 28 review records on the 600 admissions in the ePAMS+ intervention period of the study, contributed data on documentation of stopping of therapy, review of therapy or decision-making, indicating limited adoption of the review component of the ePAMS+ intervention during the trial.

Data relating to usage of the online educational material were extracted, including information on the professional discipline of the user, time spent on training and the pre- and post-test scores. Only 11 such sessions of training were logged, indicating little uptake of this form of training by the many prescribing staff in the 14 participating wards.

Table 4 summarises the DDD co-primary outcome and secondary outcomes recorded in the trial, for the periods before and after the introduction of the ePAMS+ intervention. Intravenous and oral antibiotics were used to a similar extent in the wards studied. Broad spectrum antibiotics were far more commonly prescribed than narrow spectrum ones (1725 versus 330 admissions). A typical admission had a mean of 1.6 antibiotics prescribed, for 3.4 courses on average. Treatment switches (either of route of administration or from narrow to broad spectrum antibiotic) occurred frequently. Use of Cerner EPMA system routine data as the basis for data extraction meant there were no missing data or records excluded from the analysis.

**Table 4:** Summary of outcomes by study period

	**Before ePAMS+ **		**After ePAMS+ **	
**Co-primary outcomes**				
Antibiotic administration (DDD)	*N* = 1256	3.3 (1.0, 8.5)	*N* = 587	4.0 (1.0, 9.6)
30-day mortality	*N* = 1323	111 (8.4%)	*N* = 336	36 (10.7%)
**Secondary outcomes**				
Intravenous antibiotic (DDD)^c^	*N* = 741	2.2 (1.0, 5.0)	*N* = 385	2.4 (1.0, 6.1)
Oral antibiotic (DDD)^d^	*N* = 889	3.0 (1.0, 7.4)	*N* = 402	2.8 (1.0, 7.6)
Broad spectrum antibiotic (DDD)^a^	*N* = 1170	3.2 (1.0, 7.4)	*N* = 555	3.9 (1.0, 8.7)
Narrow spectrum antibiotic (DDD)^b^	*N* = 228	2.3 (1.0, 8.0)	*N* = 97	2.5 (1.0, 11.0)
Length of hospital stay (days)	*N* = 1256	7.0 (3.3, 15.4)	*N* = 587	7.1 (3.2, 14.5)
Days of therapy	*N* = 1256	3 (1, 6)	*N* = 587	3 (1, 7)
Days of intravenous therapy^c^	*N* = 741	3 (1, 5)	*N* = 385	3 (1, 6)
Number of antibiotics prescribed	*N* = 1256	1.57 (0.85)	*N* = 587	1.67 (0.94)
Number of antibiotic courses	*N* = 1256	3.23 (2.32)	*N* = 587	3.67 (2.94)
Number of iv to oral switches^c^	*N* = 741	1.01 (2.13)	*N* = 385	1.17 (2.55)
Number of oral to iv switches^d^	*N* = 889	0.62 (1.90)	*N* = 402	0.90 (2.50)
Number of switches to alternative antibiotic	*N* = 1256	1.32 (1.59)	*N* = 587	1.58 (1.99)
Number of switches from narrow to broad spectrum^b^	*N* = 228	1.62 (2.86)	*N* = 97	2.47 (5.06)
Number of antibiotic discontinuations	*N* = 1256	1.91 (1.58)	*N* = 587	1.99 (1.83)

Summary data based on the *N* = 1843 completed admissions in the trial data set. DDD, defined daily dose. Antibiotic administration data summarised per admission. Mortality data are presented as N (%). Continuous variables reported as median (lower quartile, upper quartile) for DDD, length of hospital stay, days of therapy and days of intravenous therapy; all other continuous variables reported as mean (standard deviation). Narrow and broad spectrum antibiotics were classified according to information in the British National Formulary entry for each antibiotic [34].

^a^for admissions in which there was at least one administration of a broad spectrum antibiotic

^b^for admissions in which there was at least one administration of a narrow spectrum antibiotic

^c^for admissions in which there was at least one administration of an intravenous antibiotic

^d^for admissions in which there was at least one administration of an oral antibiotic

Following log-transformation, analysis of the antibiotic DDD per admission co-primary outcome using a normal linear model demonstrated considerable admission-to-admission variability (objective 10) in levels of antibiotic prescribing. Effects of ward, sex of the patient and ePAMS+ intervention collectively explained a minority of the variability (model R^2^, 40.1%). A factor for seasonal effects was not included in the model due to the short period of data collection. The residual variance from the model of 1.086 (on the log-transformed scale) will inform the statistical power calculation for a future large-scale evaluation of the effectiveness of the ePAMS+ intervention.

Due to the time lag in data transfers, occurrences of adverse events could not be monitored in real time. The overall number of deaths recorded in the 30 days following admissions (147 of 1659; 8.9%) was consistent with the level expected in the participating wards and was stable between the periods before and after the ePAMS+ intervention.

### Fidelity index

Although the development of the Fidelity Index (objective 6) was not fully supported by the data extract from the Cerner EPMA system due to very few uses of ePAMS+ order sets, its exploration helped us understand ePAMS+ prescribing structure and identify those aspects of intervention fidelity that are critical for decision-making by prescribers when applying ePAMS+ core principles. These critical decision-making points to assess intervention fidelity are outlined in Supplementary Table S2.

The data extract helped us define proxy measures (often date and time) linked to other variables to determine the fidelity with which intervention was delivered. For example, microbiology results could be linked using date/time as proxy to assess if these tests were ordered at the time of initial prescription and/or at review. Diagnostic confidence and other key fidelity indicators were not fully explored due to very limited documentation of use of the ePAMS+ review tool (28 review records in 600 admissions, 0.047 records per admission). Data extracts did not contain information on clicks to external links of ePAMS+ guidelines or training to assess if these were consulted during the prescribing process. As clicks to website links from Cerner are handled by generic user accounts, we would be unable to assess if these had an impact on the prescribing practice of individuals. Similarly, it was not possible to extract data for the rationale behind the action taken by the prescriber after antibiotic review, as this is currently a free text data field in the system.

## Discussion

### Summary of findings

This feasibility trial of the ePAMS+ intervention largely addressed its objectives (Table 1), despite tremendous pressure on the NHS at the time of the trial, with the study site at one point being subject to OPEL 4 measures. Similar pressures on the health service will likely occur in the future, and therefore implementation of any intervention must continue to take account of this complex environment.

The qualitative findings highlighted aspects of the ePAMS+ intervention, its promotion and training that were acceptable, although some features will need further development before wider deployment. Clinicians appreciated the availability of functionality to support antibiotic review, even on wards where antibiotic prescribing and reviewing were rigorously monitored. The ePAMS+ intervention provided an opportunity for reflecting on the patient’s entire antibiotic journey. The ePAMS+ system provided relevant information for the clinician in one place, and, effectively, brought together the frequently changing array of clinicians involved in prescribing and reviewing a patient’s prescription into one technologically-mediated space. Several factors contributed to the low uptake of ePAMS+ . Its launch was not widely promoted and the training was informal and ad hoc. Engagement with the formal online training was minimal. The use of ePAMS+ was optional, and many busy clinicians therefore simply did not use it. Prescribers identified areas where the ePAMS+ intervention did not match their practice and this configuration of ePAMS+ did not cover clinical specialties with complex antibiotic prescribing.

We gathered quantitative trial outcome and process measures from the routinely collected data held within the Cerner EPMA system. Data completeness was high for the variables extracted. We modelled variability in the key outcome measure, total antibiotic DDD per admission, indicating feasibility of this method for data collection in future research evaluating ePAMS+ . We confirmed, through the small number of antibiotic review records extracted, the low levels of ePAMS+ use within wards. Improvements to data extraction should focus on gathering antibiotic indication data, to enable measurement of outcomes such as the number of antibiotic courses for the same indication.

The Fidelity Index component of the research was able to identify the critical decision-making points for prescribers relating to ePAMS+ intervention fidelity and to develop proxy measures for these in the EPR data. However, due to very few uses of ePAMS+ order sets in the current data extract, we could not achieve the further aims of developing a scoring scale for quantifying each critical decision-making point and specifications for its automation within the EPMA system.

### Findings in the context of the existing literature

ePAMS+ is intended to build on the foundation laid by ARK [14] (Antibiotic Reduction and Konservation), the antibiotic review kit of which increased the number of timely reviews of antibiotic prescriptions by 8%, to 99%, and the number of antibiotic prescriptions stopped promptly by 26%, to 35%. ePAMS+ added a technology-based CDS component integrated within an EPMA system to the organisational and behavioural elements already present in ARK, since it was recognised [26] that without targeted adaptations EPMA systems do not necessarily facilitate improvements in AMS indicators. ePAMS+ also extends the scope of AMS support to target a wider range of possible actions at antibiotic review than ARK, which focuses on the stop decision.

As the feasibility trial findings have clearly shown, the potential benefit of adding the EPMA system-embedded CDS in ePAMS+ is accompanied by a further suite of implementation hurdles (Table 1) to be negotiated in the further development and roll-out of ePAMS+ . This is concordant with the findings of a qualitative synthesis of systematic reviews of digital AMS interventions [27]: while a benefit on AMS indicators was shown across a diverse range of digital interventions, the evidence for benefit on clinical outcomes was mixed and important sociotechnical dimensions of implementation have not yet been thoroughly evaluated. Of particular importance in this respect are interprofessional relationships, workflows, and integration and interfacing [28–30].

### Strengths and limitations

Our feasibility study demonstrates that early mixed-methods evaluation of intervention implementation can highlight where and how things are not going as planned and how these may be mitigated in future. Longitudinal elements allowed us to understand existing processes and how the intervention changed these [31]. We studied real time implementation of an ePrescribing intervention in a hospital experiencing extreme post-SARS-CoV-2 pressures of bed shortages and risks to patient safety. It provided empirical insights into real-world challenges impacting the effectiveness of the ePAMS+ implementation and gave insights into ways these could be addressed going forward. Due to these strong competing pressures in the hospital, we were unable to observe and capture the thoughts of clinicians prescribing in real time, which would have given further insight into informal practices impacting on prescribing and reviewing processes.

One limitation of the trial was that it took place in a single NHS Trust, constraining learning about intervention implementation across different care contexts. We partially offset this weakness by expanding the number and range of wards included to explore a variety of care settings, but nevertheless feasibility was evaluated in only two hospitals and contexts may be very different elsewhere. Also, observations were undertaken opportunistically as and when potential participants were available and willing to be observed which may lead to observation bias. Steps need to be taken to reduce as far as possible observation bias in any future ePAMS+ implementation study.

The feasibility nature of the trial means that conclusions cannot be drawn regarding the effectiveness of ePAMS+ . We also studied insufficient sites to quantify the level of clustering by site in prescribing outcomes, which, if available, would inform the design of a future effectiveness trial of ePAMS+ . The low level of uptake of the intervention and the qualitative insights gained provide a clear indication of the changes required to enable effectiveness of ePAMS+ going forward.

### Implications for ePAMS+ development

Although the interviews with prescribers showed that the ePAMS+ technical tool was broadly acceptable, it is clear to the clinical leads in the research team that to ensure meaningful adoption of the intervention in future evaluations there must be also be a switch from optional to mandatory use, once the required amendments to ePAMS+ signalled by the feasibility trial findings have been implemented. In making this recommendation we recognise the inherent difficulties in evaluating an intervention with a tightly defined scope in a complex environment with multiple patient groups, working processes and prescribing contexts.

Table 5 provides more detailed changes to ePAMS+ prompted by the feasibility trial findings and the changed context of prescribing practice and healthcare delivery following the SARS-CoV-2 pandemic. Priority technical tool changes to integrate ePAMS+ better in existing workflows include, for example, including commonly used combinations of antibiotics in the list of available ePAMS+ order sets, and making ePAMS+ readily accessible from the Medications section of the EPR rather than its current less intuitive position under Request and Care Plans.

**Table 5:** ePAMS+ design changes in response to feasibility trial findings

Feasibility trial ePAMS+	Future ePAMS+ design
**TECHNICAL COMPONENTS**	
ePAMS+ optional	ePAMS+ compulsory
Antibiotic order through Requests and Care Plans	Antibiotic order to appear on new Medications list
* Not included*	Supporting orders included e.g. cultures
* Not included*	Hyperlink to guidelines
* Not included (single antibiotics only)*	Include most common antibiotic protocols/order sets including antibiotic combinations
* Not included*	Hyperlink to revised training
Antibiotic review	Antibiotic review more accessible
*Not included – (pop-up reminder for review [lockdown] due to safety concerns)*	
* Not included*	Adult discharge / outpatient ePAMS+
* Not included*	Paediatric inpatient ePAMS+
* Not included*	Paediatric discharge / outpatient ePAMS+
* Not included*	Hide antibiotic orders on non-ePAMS+ Medications list
* Not included*	Text box for antibiotic review narrative
**NON-TECHNICAL ELEMENTS**	
Not included	Project manager and trainers / floor walkers
Online training	Online AMS training
Ad hoc training	Face to face training compulsory
* Not included (competing priorities)*	Patient Information Leaflet
* Not included (competing priorities)*	Clinical discussion groups
Ad hoc engagement / launch plan	Formal engagement / launch plan

The launch of the ePAMS+ intervention did not go as planned due to hospital pressures at the time. This was a major limitation and needs to be addressed in any future implementation of an amended ePAMS+ intervention.

Regarding AMS and ePAMS+ training, there was a reported preference for learning to be on the job or added to other mandatory training or study. Although this sounds feasible, an additional tool for training may be required for wider roll out of system change, to ensure a broad audience can be reached in a short space of time. This could then be supported by in-practice informal training.

Finally, further development of a Fidelity Index to support future evaluations of ePAMS+ will require a dataset including several hundred examples of antibiotic reviews, which would be gathered via piloting of the updated ePAMS+ technical tool.

### Implications for policy and further research

Despite the diverse range of ward types studied in this trial, wider generalisability of these findings to the other NHS Trusts using Cerner and to Trusts which have adopted alternative EPMA systems is unclear. The next priority will therefore be to extend piloting of the updated ePAMS+ intervention to a broader range of contexts, in recognition of the known variation in the functionality of Cerner and other EPMA systems across NHS Trusts. We did nevertheless gain insights into some implementation and adoption challenges associated with AMS-based ePrescribing functionality. Future evaluations of ePAMS+ will also need to consider a broader range of outcomes than antibiotic DDD and mortality, incorporating days of therapy and a range of process of care measures to enable the impact of ePAMS+ on the quality of prescribing to be assessed fully. Within the future ePAMS+ evaluation, a validation of the psychometric properties of the Fidelity Index will be undertaken [32].

The extreme circumstances in which the feasibility trial was undertaken provide important lessons for the roll-out of interventions – typically understood by suppliers of digital healthcare innovations in an idealised manner as an introduction of change on a “blank slate” [33]. In reality, healthcare organisations are socio-technical systems with embedded managerial and technical activities that have formed over time, influenced by previous technological and organisation-level changes [33]. The kinds of acute pressures experienced, which frustrated plans for coordinated training and awareness exercises alongside ePAMS+ implementation, are likely to be a repeated feature of health service implementations in times of economic crisis.

This feasibility trial has highlighted the value of early mixed-methods evaluation of a technological intervention and, just as importantly, of the implementation process. In particular, a timely qualitative evaluation will (1) determine the need for further intervention development to meet clinicians’ and patients’ needs; (2) establish how the intervention fits into clinicians’ workflow and any workarounds they have developed; (3) refine ways of implementing the intervention to promote adoption; and (4) identify early signals of benefit or unintended consequences of the intervention.

## Conclusions

Whilst it is important to have a person-based approach to intervention development, real-world implementation may encounter circumstances unforeseen by stakeholders due to contextual factors, including external influences such as the effects of a global pandemic and other capacity and workload pressures within the NHS. Furthermore, no intervention is implemented in a static environment: this needs to be accounted for when designing implementation strategies and carefully adapting these to local circumstances, which in healthcare are complex, diverse and constantly changing. Therefore, implementers need to proceed flexibly, open to the possibility of changing plans to achieve the ultimate benefits for clinicians and patients.

These feasibility trial findings also offer a detailed series of action points to inform refinements of the ePAMS+ intervention and guide the plans for its future evaluation, and ultimately adoption in clinical practice. Consequently, we conclude that before progression to a confirmatory effectiveness trial, further piloting of the updated intervention and its accompanying implementation plan, in a range of different care contexts, will be required before the goal of supporting important improvements in AMS through ePAMS+ can be realised.

## Supplementary Information

Supplementary Material 1.

Supplementary Material 2.

Supplementary Material 3.

## Data Availability

De-identified quantitative data for this trial are held within the Scottish National Safe Haven, having been obtained from routinely collected health data within the Cerner EPMA system. These data are not appropriate for public sharing, as consent was not sought from eligible admissions in this service-level evaluation of intervention feasibility. The qualitative datasets generated and/or analysed during the current study are not publicly available to protect the anonymity of participants, but are available from co-author KC on reasonable request.

## Abbreviations

AMR: Antimicrobial Resistance
AMS: Antimicrobial Stewardship
CDS: Clinical Decision Support
CPOE: Computerised Provider Order Entry
CQUIN: Commissioning for Quality and Innovation
ePAMS+: EPrescribing-based Anti-Microbial Stewardship intervention
EPMA: EPrescribing and Medicines Administration
EPR: Electronic Patient Record
NHS: National Health Service
OPEL: Operational Pressures Escalation Level
SARS-CoV-2: Severe Acute Respiratory Syndrome Coronavirus 2
UK: United Kingdom
