# Supplementary material for: A complex ePrescribing antimicrobial stewardship-based (ePAMS+) intervention for hospitals: mixed-methods feasibility trial results
Source: BMC Med Inform Decis Mak. 2024 Oct 11;24:301. doi: 10.1186/s12911-024-02707-9 (PMC11470576; doi:10.1186/s12911-024-02707-9)
Supplement: Supplementary file 1 — Supplementary Material 1. [file 12911_2024_2707_MOESM1_ESM.pdf]

Topic Guide Healthcare Professionals Interviews

Introduction (used for all interviews)

- Thanks a lot for taking part. The interview should take no longer than 60 minutes.
- Brief reminder of the aims
- Do you have any questions about the study or do you feel the information you received is sufficient?
- And you are still ok with the interview being audio-taped and transcribed?
- The transcripts will be anonymised, anything you say during the interview will be confidential, you can ask for the interview to be stopped at any time

Please note: Topic guides will be used as guidance only and not in all interviews all of the topics may be covered as participants will be encouraged to raise issues important to them. Also, we will include follow up questions to explore the different aspect of ePAMS+.

Brief description of the goals of the intervention and WP1:

- Public Health professionals are seeking to mitigate risk that uncontrolled prescribing will increase prevalence of antibiotic resistant infections
- We are part of a project (ePrescribing-based Antimicrobial Stewardship (ePAMS+) that is exploring whether information tools can improve prescribing practices for antibiotics (described as Anti-Microbial Stewardship AMS)
- This work package will first optimise the design of our computer-based Anti-Microbial Stewardship intervention and then assess its impact on prescribing practices and patient health

Questions

Background and involvement in ePAMS+ (What involvement do you have in antimicrobial use in the hospital (e.g. prescriber, administration of antibiotics, educating other healthcare professionals, advising staff etc.))

In your opinion, what are the main issues with the prudent prescribing of antimicrobials in general and (if different) specifically within this institution?

What is your understanding of the intervention?

What are your expectations of this project? (What do you think it will achieve/lead to?)

In your opinion, what are the positive and negative aspects of ePAMS+? (Any concerns/problems? What aspect is running particularly smoothly/effectively? Why?)

What do you feel about the outcome measures that have been chosen?

How well did ePAMS+ promote antimicrobial stewardship in your hospital? (If it works, why do you think this will be? If it doesn’t work, why do you think this will be?)

In your opinion, how does the intervention affect work flow, processes and relationships of healthcare professionals and the patients they look after?

How might the design/usability/intelligibility of the intervention be improved?

Is there anything that you would have done differently in implementing ePAMS+?

If ePAMS+ is shown to be effective, what do you think would help to get it utilised on a larger scale and effective? (Across England)

How do you think organisations/staff can be motivated to implement interventions like ePAMS+?

Is there anything else that you would like to say?

Anyone else you think we could talk to/meet?
