# Supplementary material for: A complex ePrescribing antimicrobial stewardship-based (ePAMS+) intervention for hospitals: mixed-methods feasibility trial results
Source: BMC Med Inform Decis Mak. 2024 Oct 11;24:301. doi: 10.1186/s12911-024-02707-9 (PMC11470576; doi:10.1186/s12911-024-02707-9)
Supplement: Supplementary file 2 — Supplementary Material 2. [file 12911_2024_2707_MOESM2_ESM.docx]

**Supplementary Table S1 Qualitative study themes and quotations**

| **Theme** | **Quotations** |
| --- | --- |
| **Promoting antibiotic review** | *“I mean everywhere's busy, but they're busy in different ways and I think in those instances it might be useful just to have that reminder and that can be a moment where you can actually just think, is this the right antibiotic? And then check the guidelines, et cetera. And so I think …there's definitely a good case for it”. (Participant 3, junior doctor, foundation year 2)*  *“The ePAMS system lets you document things as you’re going along, so you might have a senior review but you might not have the blood culture results back yet and various things like that. So, it does provide a framework for ticking those things off, as it were…I think in the [specialty], there has always been quite a high awareness of all those kinds of things but yes, there has not been that formal system in place to essentially force somebody to tick that they have seen the blood culture results and that they have had a senior review about it and things like that.” (Participant 1, consultant)*  *“Well, yeah, it's much better for your antimicrobial stewardship, and personally…because I've worked on the wards, I would be checking antibiotics daily anyway to see if they're ready for an oral switch. But I think to prompt people so that they don't miss it is a good idea, because obviously there are days when you don't have the opportunity to be so thorough or you just miss it because you're doing something else. So definitely a prompt is useful.” (Participant 22, junior doctor, foundation year 2)*  *“I think it’s definitely great in that it prompts you to do a medication review and it prompts, you know, especially on places like this ward in particular, with our rota, where there’s not always that continuity of juniors covering the same service each week or even day to day, it definitely prompts juniors later on in the week, to review those medications and look at them.” (Participant 8, junior doctor, foundation year 2)* |
| **Training and launch of ePAMS+** | *“So on the assessment suite, we have teaching for 15 minutes every morning, so then as one of the teachings, he* [consultant lead] *came in, was like explaining why antimicrobial stewardship’s important and then said, can we all prescribe antibiotics using ePAMS, so that they get a review within 48 hours.” (Participant 15, speciality trainee, year 2)*  *“It requires raising awareness about a new electronic system is, in and of itself, a separate project. So, it's great that we have that now going, but it's a lot of work…to raise awareness and encourage clinicians to use it. And then once you do that, you still need to redo the awareness project every few weeks, really, on the assessment suite, as trainees change every few months, as the trainees rotate around hospital…So, it's a whole culture change…” (Participant 5, specialty trainee, year 5)*  *“In my head I’m trying to think how it would last half an hour, because, you know, [Lead Consultant] came down, explained the premise and showed us how to do it in about five, ten minutes, so I don’t really see why it needs to be longer …I think some training on the review would be useful. Even just…I don't think it would need to be particularly like in-depth. Like even just a five or ten minute session. Ideally maybe added on to some pre-existing teaching, I don't know, so the foundation year, doctors' teaching or the IMT [Internal Medicine Trainee] teaching, I think would be useful.” (Participant 24, specialty trainee year 2)*  *“I had [Consultant 1] explain it to me on the way there last week, and then [Consultant 2] came down, because…to teach the other F1, so that was like a mini refresher, but I was busy doing jobs and sort of casting an eye over as well.” (Participant 15, speciality trainee, year 2)* |
| **ePAMS+ user experience** | *“Usually when I'm prescribing antibiotics, I'm very busy because I'm reviewing unwell people at the admissions unit. Because that tends to be when they're started on them. And because I've been prescribing them for three years without using ePAMS, I have to really force myself to remember and I honestly don't always.... But I think it's reasonably easy to use otherwise.” (Participant 21, speciality trainee, Year 1)*  *“It’s no more bother for me to prescribe it via ePAMS than to prescribe it normally…it’s just as easy, and then if it helps further down the line to stop inappropriate use of antibiotics…” (Participant 15, speciality trainee, Year 2)*  *“I think it’s a good idea. I definitely agree with the principle of it, but I think it’s annoying that if…so normally if I’m prescribing on eRecord I’d go into ‘Medication’ list and then click ‘add’ and then everything is through there but if I try to do ePAMS via that, it doesn’t work so I have to go into this ‘Requests and Care plans’ bit and add it through there.” (Participant 8, junior doctor, foundation year 2)*  *“So, I think these order sets, I think in my career, I’ve just found them quite helpful in that they prompt you and then it’s just a human factor, they just prompt you and it reduces…also I think ePAMS would be really good if ePAMS had order sets in the same way.” (Participant 10, junior doctor, foundation year 2)*  *“When the ePAMS plan comes, it gives you a kind of suggested dose… and probably the suggested dose for each of the antibiotics needs to… be the one we would want people to prescribe most commonly, and that’s not quite the case at the moment, so that just needs tweaking”. (Participant 17, consultant)*  *“The only difficulty is if you need to prescribe, say, two antibiotics at once, like amoxycillin and clarithromycin, you’ve got to do one, sign it off, and then do the other. For some reason, it won’t let you select two at once.” (Participant 19, specialty trainee, year 3)*  *“So, actually you have to do it a different way, which is you have to …go to ‘Requests and Care Plans’ and get rid of the plan… then you have to prescribe everything from scratch again….It’s a lot of work, it’s just more work than necessarily than needs to be, I’m being a bit dramatic but it’s an extra four or five steps that need doing.” (Participant 20. specialty trainee, Year 1)* |
| **Integration of ePAMS+ with multidisciplinary ways of working** | *“It’s really time-consuming doing IV, intravenous. It can be detrimental to the patients because they’ve got…you’re going into a vein, there’s risks of getting infections from cannulas or midlines so there is a risk to that and it’s more intervention than what you would do taking an oral medication. On a timescale, we’re preparing nearly all day for IVs because it’s from breakfast time and we’ve got 18 patients and sometimes people have two or three IVs. You could be doing 30 odd IVs drawn up. It takes you so long, so we like to keep an eye. We know that when we can swap over from IV to oral, we’re just popping a pill out of a pot. Whereas we’re having to mix it, go and administer it, manage the patient’s cannula and then also going back and disconnecting, flushing the line, making sure the line’s patent and there’s no sign of infection so it’s a big part of our day doing IV administration.” (Participant 5, senior nurse)*  *“I think anything that’s got to be put about and advertised and brought up, it’s often done by the nurses. I think the doctors are a little bit more, if it’s left to them, it doesn’t always get done. It’s just I think they’ve got quite a lot on so they don’t always get round to doing things like that.” (Participant 5, senior nurse)* |
