# Supplementary material for: A complex ePrescribing antimicrobial stewardship-based (ePAMS+) intervention for hospitals: mixed-methods feasibility trial results
Source: BMC Med Inform Decis Mak. 2024 Oct 11;24:301. doi: 10.1186/s12911-024-02707-9 (PMC11470576; doi:10.1186/s12911-024-02707-9)
Supplement: Supplementary file 3 — Supplementary Material 3. [file 12911_2024_2707_MOESM3_ESM.docx]

**Supplementary Table S2 Key data capture points to assess intervention fidelity for ePAMS+**

| **Fidelity data capture point** | **Variable/s in the data extract** |
| --- | --- |
| 1. Whether antibiotics were ordered individually, using ePAMS+ order plan or alternative order plans | PATHWAY_CATALOG_ID |
| 1. Indicator variable to describe if antibiotic prescription was informed by ePAMS+ principles as intended:    1. Labs/cultures ordered    2. Duration prescribed    3. Guidelines consulted | Index to be constructed using variables 2a – 2c:  Date and time variables as proxy measures to link with prescription  Calculated from start and stop date/time variables  Currently not possible to extract data on clicks to external links |
| 1. Use of decision aid (data on diagnostic confidence, whether indicated and what level was chosen) | PATHWAY_CATALOG_ID (needs to be mapped to decision aid categories before use) |
| 1. Indicator variable to describe if antibiotic review was informed by ePAMS+ principles as intended:    1. Whether prescribed antibiotic was reviewed    2. Timing of the review    3. What was reviewed when making decision (e.g. clinical diagnosis/lab results)    4. Decision taken (stopped, switched, changed or continued)    5. Rationale for the decision taken | Index to be constructed using variables 4a – 4c:  OA_ACTION_TYPE_DISP  Calculated from date/time variables  Free text data that will need coding and categorising when reasonable ePAMS+ usage data is available  OA_ACTION_TYPE_DISP; Date/time stamp to be used as proxy to determine whether the review led to an action  Not possible to extract as open-ended field in the system |
| 1. Temporal association of critical decision points with actions taken | Proxy measure of Review completion with Encounter ID and data/time stamp (*note there can be many encounter IDs for each patient*) |
